# An ${\text{ACO}}_{R}$-Based Multi-Objective WSN Deployment Example for Lunar Surveying

**DOI:** 10.3390/s16020209

**Published:** 2016-02-06

**Authors:** Pablo López-Matencio

**Affiliations:** Information and Communications Technologies Department, Technical University of Cartagena (UPCT), Cartagena 30202, Spain; pablo.lopez@upct.es; Tel.: +34-968-325-977

**Keywords:** WSN, deployment, multi-objective, ACO, optimization, moon

## Abstract

Wireless sensor networks (WSNs) can gather *in situ* real data measurements and work unattended for long periods, even in remote, rough places. A critical aspect of WSN design is node placement, as this determines sensing capacities, network connectivity, network lifetime and, in short, the whole operational capabilities of the WSN. This paper proposes and studies a new node placement algorithm that focus on these aspects. As a motivating example, we consider a network designed to describe the distribution of helium-3 (${}^{3}$He), a potential enabling element for fusion reactors, on the Moon. ${}^{3}$He is abundant on the Moon’s surface, and knowledge of its distribution is essential for future harvesting purposes. Previous data are inconclusive, and there is general agreement that on-site measurements, obtained over a long time period, are necessary to better understand the mechanisms involved in the distribution of this element on the Moon. Although a mission of this type is extremely complex, it allows us to illustrate the main challenges involved in a multi-objective WSN placement problem, *i.e.*, selection of optimal observation sites and maximization of the lifetime of the network. To tackle optimization, we use a recent adaptation of the ant colony optimization (${\text{ACO}}_{R}$) metaheuristic, extended to continuous domains. Solutions are provided in the form of a Pareto frontier that shows the optimal equilibria. Moreover, we compared our scheme with the four-directional placement (FDP) heuristic, which was outperformed in all cases.

## 1. Introduction

Fusion is attracting increasing attention, as unlike fission reactions, fusion reactions do not generate radioactive waste. Fusion reactors are designed to mimic nuclear reactions produced in the Sun by forcing together the nuclei of two hydrogen isotopes: tritium and deuterium [1]. The byproducts are energy, helium and high-energy neutrons, which are a containment risk [1,2]. This obstacle, however, can be overcome by substituting tritium for helium-3 (${}^{3}$He). ${}^{3}$He is a single neutron isotope of helium produced naturally through hydrogen fusion in the Sun. Unfortunately, the Earth’s atmosphere and its magnetic field repel this element, and only traces of this element exist on Earth. The Moon, by contrast, has accumulated large amounts of ${}^{3}$He on the uppermost layer of its surface [3] (lunar regolith), bringing researchers on Earth to consider the possibility of mining this element from the Moon [4,5,6].

An endeavor of this magnitude, however, would require detailed planning and, of course, knowledge about the distribution and abundance of ${}^{3}$He on the Moon. Estimations of ${}^{3}$He abundance and distribution rely on parameters, such as solar wind flux, lunar regolith composition and maturity, lunar regolith grain size and regolith thickness. The compilation of all of these variables in a system model makes ${}^{3}$He estimation an extremely complex task. Indeed, existing ${}^{3}$He distribution and reserves models are marked by critical discrepancies due to imprecise or insufficient lunar data [7]. More accurate estimations would require large-scale, more detailed on-site surveying. Recent studies by Prasad and Murty [8], Pabari *et al.* [9] and Zhai *et al.* [10] have brought attention to the potential of wireless sensor networks (WSNs) for geological and mineral analysis on the Moon. In this paper, we focus on this scenario as a motivating example to evaluate a novel algorithm for optimal WSN placement.

WSNs are considered an enabling technology for unattended, long-lasting and rough terrain monitoring and have been widely studied in recent years [11,12]. They are constituted by multiple, small, relatively inexpensive nodes, which gather and relay environmental data to sink nodes, which in turn, forward this data to control centers. In any realistic WSN deployment, the placement of nodes must be carefully planned to ensure they are located in the best possible observation sites and to maximize the quality of the information gathered. In our proposed lunar ${}^{3}$He survey, it is critical to select sites that would improve the chances of obtaining trustworthy information on ${}^{3}$He distribution and abundance. Such a decision would normally be made on the basis of previous studies that have either directly or indirectly assessed the quality of each site. In our case, however, there are no maps available that directly characterize ${}^{3}$He distribution, although in recent works, authors, such as Li *et al.* [7], Slyuta *et al.* [13], WenZhe and YaQiu [14] and Zheng *et al.* [15], agree that there might be a direct correlation between TiO${}_{2}$ and ${}^{3}$He abundance. In Section 5.1 of the paper, we therefore consider a two-dimensional target area in which non-uniform mapping of TiO${}_{2}$ indicates the suitability of each site for ${}^{3}$He characterization. These maps can either offer a finite set of candidate sites (discrete placement) or place no constraints on placement (continuous placement). We focus exclusively on the latter case, since discrete placement is much simpler and has already been studied in depth [16,17].

Maximal spatio-temporal resolution is also desirable in geological surveys. On the one hand, this implies minimal separation between nodes to avoid redundant information, and on the other hand, it places a limit on maximal separation, as nodes need to establish radio-communication links with each other. In general, placement must guarantee network connectivity, *i.e.*, it must guarantee the establishment of routes for conveying information to the sinks. Both questions are considered in our lunar example: minimal separation is addressed by using nodes with a sensing range, while connectivity is a constraint imposed on the problem.

Energy is also a major consideration in WSN placement. Access to battery replacements may be cost-prohibitive (e.g., polar surveys) or even impossible (e.g., a lunar survey) in hostile environments. Alternative sources, such as solar panels, are not always feasible (a lunar night, for example, lasts ∼14 Earth days), but may offer chances for recharge. For our scenario, energy efficiency should be explicitly included in the design of the WSN to prevent battery outages. Naturally, however, this goal must be balanced against another major concern: data quality. The bulk of WSN energy is consumed during radio communications, *i.e.*, during transmission and reception (either of actual messages or idle listening). Therefore, we consider that energy consumption should be determined by the amount of data generated by each node (directly related to the quality of the site) and the “length” of the path (in hops) to the sink.

In brief, our proposed optimization scheme has two objectives: (i) to maximize the chances of obtaining good ${}^{3}$He distribution data and (ii) to minimize energy consumption. Finding exact solutions to problems of this type can be a daunting task given the complexity and, in many cases, may even become computationally intractable [18]. One solution chosen by many authors to find near-optimal solutions to multi-objective deployment problems is the use of metaheuristic algorithms (see Section 2), which is very much still an open research area. In our case, we drew on research by Socha and Dorigo [19], which extends the ant colony optimization (ACO) metaheuristic (see Dorigo *et al.* [20]) to handle continuous domains. This algorithm is termed ${\text{ACO}}_{R}$, and to our knowledge, this is the first time it has been applied to a WSN deployment problem. Our approach is intended to be more realistic than previous, related studies in several respects:

(1) The proposed deployment model considers coordinates as continuous variables.
(2) We explore the novel use of the ${\text{ACO}}_{R}$ metaheuristic in a deployment problem and a multi-objective optimization problem. The literature on deployment problems in relation to ACO is limited, and we are faced thus with an open area of research. In addition, our work is the first ACO-related research to use preferential sensing coverage.
(3) We present an original application scenario. Multi-objective deployment problems to date have mostly been applied to small-gridded, artificially-developed scenarios (see Section 2). We, by contrast, test our approach on a large extension of the Moon surface containing traces of ${}^{3}$He: the Dionysius region.
(4) We have adjusted parameters of the ${\text{ACO}}_{R}$ algorithm to the deployment problem. This procedure and the resulting optimization model could be extended to other target scenarios and optimization objectives.
(5) We evaluated the proposed deployment methodology by comparing ${\text{ACO}}_{R}$ to a simple heuristic in terms of coverage. A tradeoff between joint-coverage and energy cost is also computed, which could be useful when planning a lunar exploration mission.

The remainder of this paper is organized as follows. Section 2 introduces related work. Section 3 presents the deployment problem and formulates it as a multi-objective optimization problem. Section 4 overviews the ${\text{ACO}}_{R}$ algorithm. Section 5 describes our target scenario and the results achieved. Finally, Section 6 concludes the paper.

## 2. Related Works

In-depth reviews of the use of metaheuristics for WSN deployment problems are presented by Deif and Gadallah [16] and Tsai *et al.* [18]. In the latter case, the authors differentiate between metaheuristic algorithms that look for a solution in one and only one direction (in multi-variable space) at each iteration, such as tabu search or simulated annealing, and metaheuristics capable of searching in more than one direction at a time (*population-based algorithms*). These include evolutionary algorithms and swarm intelligence, which we focus on in the next section.

Swarm-based algorithms have been used to address multi-objective optimization deployment problems in recent years. These metaheuristics are inspired by the resilient property of certain biological species to collectively solve complex tasks (swarm intelligence). For instance, the ability of ants to find the shortest path to food inspired the development of ACO [21], while bird-flock movements during foraging led to the development of particle swarm optimization (PSO) [22]. These methods have outperformed common node placement techniques. Banimelhem *et al.* [23] and Pradhan and Panda [24], for example, use a genetic algorithm and binary particle swarm optimization method, while Liao *et al.* [25] compare virtual forces and glowworm swarm optimization, and Yu *et al.* [26] use artificial bee colony (ABC) optimization. A comparison deployment study performed by Mini *et al.* [27] shows how ABC outperforms PSO in terms of prolonging network lifetime. The paper by Sengupta *et al.* [28] introduces a fuzzy dominance-based decomposition technique in a multi-objective evolutionary algorithm that improves results obtained with PSO and simple genetic algorithms.

ACO has been widely applied to several networking problems, such as routing problems, as pointed out in the work of Kulkarni *et al.* [29]. For example, Cheng *et al.* [30] focus on network lifetime extension. Saleem *et al.* [31] deal with network hole minimization (*i.e.*, the minimization of unconnected parts), whereas in [32], the same authors focus on optimizing delay, packet loss and power consumption. Lin *et al.* [33] and Ye and Mohamadian [34], in turn, propose mechanisms to eliminate redundancy by combining data from different sources in order to improve performance. In a recent study, Liu [35] presented an optimal distance-based transmission strategy to improve network lifetime. All of these works show that ACO-based solutions provide flexible and sound routing solutions. However, until now, ACO has been barely used to address deployment problems in WSNs.

Several studies [36,37,38,39,40,41] have attempted to formulate deployment as a multi-objective *discrete*optimization problem with the application of ACO. In [36], the deployment aims to achieve full coverage in a gridded region with a minimum number of nodes. The authors employed a variant of ACO (Max-Min Ant) to solve the problem and showed that it can improve genetic-based algorithms in terms of the number of sensors. In [37], the authors presented a modified ACO algorithm to adjust the solution to different situations in the convergence process. The goal in [38] is to deploy sensors along a grid to cover points of interest while maintaining network connectivity. This procedure is similar to the local search method proposed by Rebai *et al.* [42] and to the four-directional placement (FDP) heuristic described in Section 5.1, which we use as a reference proposal. In addition, the authors use non-uniform node deployment to place more nodes in areas with a heavier load, thus increasing lifetime as a trade-off for deployment cost. By contrast, the ACO system approaches described in [39,40,41] only address optimization of network energy resources. Liao *et al.* [39] adapt ACO to solve a multi-knapsack problem, where energy is a resource that must be optimized among clusters of sensors. Anil Kumar and Thomas [40], in turn, use ACO to select optimal sub-sink nodes in order to minimize energy cost when transmitting to a mobile sink. Finally, Lin *et al.* [41] maximize network lifetime by using ACO to compute optimal clustering of the network while maintaining full coverage. None of these ACO deployment problems consider the continuous domain of node positions, or use ${\text{ACO}}_{R}$, and in addition, they all target discrete or gridded areas that, unlike our lunar example, are unrealistic.

Other non-ACO multi-objective node placement-related approaches are described in [43,44,45,46,47,48,49,50]. Like us, the authors consider a deployment area characterized by the spatial irregularity of the sensed event. The goal of works [43] and [44] is to find global Pareto-optimal solutions to a multi-objective deployment problem with coverage, connectivity and lifetime constraints. Two heuristics are proposed: an adaptation to several objectives of the tabu search metaheuristic and a virtual potential field algorithm. Sengupta *et al.* [48] use the same multi-objective optimization methodology as in [28] to achieve maximum lifetime and coverage. In this case, instead of dealing with traditional grid or uniform coverage, the authors focus on probabilistic coverage in regions that require different levels of sensing. In these works [43,44,48], tests are developed in small, artificially-generated scenarios. The authors of [45,47,50] also use small test scenarios, but in this case, they are more realistic. In [45] and [47], the authors present a novel hybrid scheme based on geostatistical analysis and the Monte Carlo technique. The aim is to find optimal sites with the minimum averaged variance of the measured phenomena. Deployment tests are performed with realistic data: mercury in soil outside Oak Ridge Reservation in [45] and chromium contamination at Los Alamos National Laboratory in [47]. None of the works [45,46,47,49] address the optimization of energy costs. One algorithm that does address energy cost is that presented by González-Castaño *et al.* [50], whose aim is to locate shots in a national park to detect poaching. Node sites are selected for maximal sensing coverage and minimal installation cost (related in this case to distance to power lines). Nonetheless, the optimization methodologies used in [45,47,49,50] are not bioinspired.

In short, the literature on multi-objective deployments using ACO is scarce. In the models described, node positions are restricted to a finite set of candidate sites (discrete optimization), and tests are undertaken in small artificial scenarios. Furthermore, none of the ACO deployment studies reviewed consider irregularities in sensing coverage. We, by contrast, tested our model for use in a large realistic deployment scenario (the Moon), where positions can be selected from a continuous set. We used ${\text{ACO}}_{R}$, a new ACO metaheuristic, for continuous variables. To our knowledge, this metaheuristic has not been used before in either single- or multi-objective deployment problems.

## 3. WSN Deployment Model

**Figure 1:**
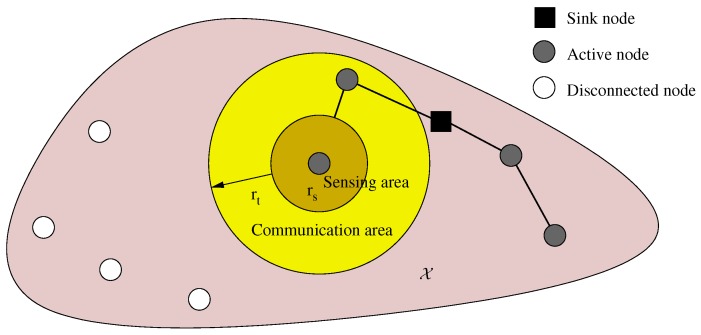
Connectivity and sensing parameters.

Our model comprises a set of *N* nodes (including a base/sink node per network). Let us recall from Section 1 that the WSN must be deployed in a target area where placement sites have a non-homogeneous quality. The goal is to maximize the importance (information about ${}^{3}$He distribution) obtained. In other words, sites expected to contain higher traces of ${}^{3}$He are more *important*than sites with lower estimations of ${}^{3}$He traces. The planning is subject to communication constraints, as well, since nodes must have a path (either direct or indirect via a multi-hop route) to the sink node. Figure 1 illustrates the main elements of the model.

**Figure 2:**
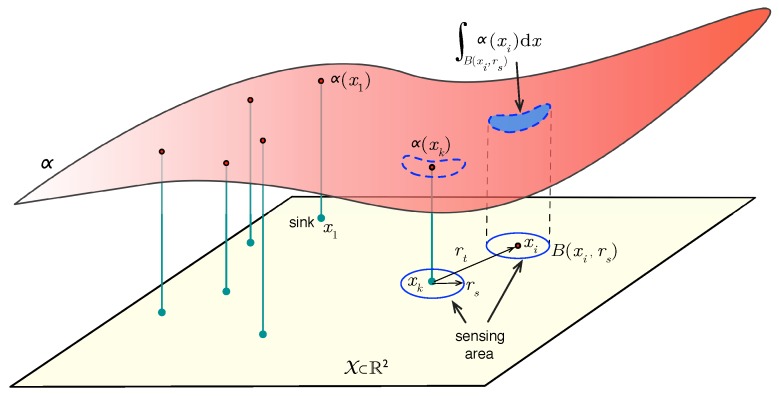
Problem description.

Formally, let a region $X\subseteq R^{2}$ represent the target area. There exists an *importance*mapping (representing expected ${}^{3}$He distribution) associated with every point x∈X, which is defined by a real function $\alpha :X\to R_{0}^{+}$. A solution $s={\left\{x_{i}\right\}}_{i=1,\ldots ,N}$ is a set containing the position of nodes, s∈S, and S is the set of all (candidate) solutions. Besides, the following considerations have been established:

- The hardware of the nodes is homogeneous, *i.e.*, it is of the same type and has the same communication/sensing capabilities.
- The sink node can be any of the *N* sensor nodes. Without loss of generality, we assume that it is Node 1 and is therefore positioned at $x_{1}$.
- The dimension of our problem is 2N, since a solution is composed of positions of *N* nodes in an $R^{2}$ space.
- The *sensing range* $r_{s}$ is the minimum significant separation required between two nodes to consider their sensing data independent. The information gathered by sensor *i* is then given by:
  (1) $$I_{i}=\int_{B(x_{i},r_{s})}\alpha \left(x\right)dx$$
  where B(x,r) is the open ball in $R^{2}$ centered in *x* with radius *r*. Figure 2 depicts this model.
- The *transmission range* $r_{t}$ is the longest separation distance between two mutually communicating nodes, and it determines network connectivity. Let us term *active* nodes all nodes able to transmit their sensed data to the sink, and let *A* denote this set of nodes.

### 3.1. Coverage Objective: Importance

If sensing areas of active nodes overlap, the information gathered is counted only once, since as stated above, it is not independent. In other words, the open set describing the sensed area is:

(2) $$B=\underset{i\in A}{⋃}B\left(x_{i},r_{s}\right)\cap X$$

Thus, the sensing is given by $f_{1}:S\to R_{0}^{+}$,

(3) $$f_{1}\left(s\right)=\int_{B}\alpha \left(x\right)dx$$

which measures the importance (*i.e.*, information about ${}^{3}$He) covered by the nodes and which we aim to maximize.

### 3.2. Energy Objective: Cost

**Figure 3:**
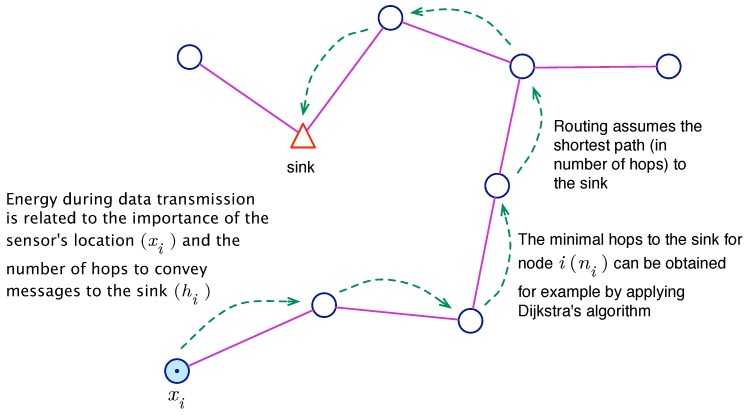
Routing of messages generated by node $x_{i}$ towards the sink.

Energy efficiency is another important factor that must be taken into account. As stated previously, it is mandatory to reduce global energy consumption. In our model (see Figure 3), we assume the shortest hop-count path routing, as well as an anti-collision layer based on a duty cycle with on/off periods. Both strategies are common in WSNs [51].

In WSNs, energy consumption is mostly due to message transmission/reception, and other sources of waste, such as CPU load or electronics sensing, have only a minor impact [52]. With the assumption of an operation in a duty cycle, nodes turn on the radio during the active stage for the transmission and reception of messages. The energy used depends on the number of transmitted messages. In our model, we reasonably assume that more important places cause more transmission events, and therefore, transmission consumption is proportional to α(x) if the node is placed at *x*. In addition, the routing of the node also plays a central role in energy consumption, since the messages must be retransmitted by intermediate nodes on their way to the sink. Let $h_{i}$ denote the number of hops on the minimal hop-count route from node *i* to the sink. Then, the energy associated with the transmission of a node *i* can be expressed as $e_{\text{tx}}\alpha \left(x_{i}\right)h_{i}$, where $e_{\text{tx}}$ is constant and represents the energy used per message (assuming constant-length messages).

Finally, the reception consumption is considered constant (let $E_{\text{rx}}$ denote it) in most WSN models [53], since radios are permanently on during the active part of the duty cycle. That is because it is not possible to forecast when incoming messages will arrive. From this perspective, an expression for energy consumption in the WSN can be obtained directly as follows:

$$f_{2}\left(s\right)=e_{\text{tx}}\sum_{i=1}^{N}\;\alpha \left(x_{i}\right)\;h_{i}+E_{\text{rx}}\;N$$

Since the goal is to minimize this function, constants $e_{\text{tx}}$ and $E_{rx}$ can be safely removed from the optimization problem description, leading to:

(4) $$f_{2}^{*}\left(s\right)=\sum_{i=2}^{N}\;\alpha \left(x_{i}\right)\;h_{i}$$

Note also that $h_{1}=0$ because Node 1 is the sink.

### 3.3. Multi-Objective Deployment Problem

Our ultimate task is to place *N* sensors such that the information covered, $f_{1}$, is maximized and the energy cost, $f_{2}^{*}$, is minimized. Clearly, both objectives are contradictory and must be balanced: function $f_{1}$ selects positions with more information about ${}^{3}$He, whereas $f_{2}^{*}$ tends to concentrate nodes close to the sink. For that reason, it is desirable to produce a Pareto front [54] for these two goals. A Pareto front is a set of non-dominated solutions and represents a pool of *candidate* optimal solutions, which allow the establishment of optimal trade-offs in the problem balance.

This joint problem can be stated as:

(5) $$\begin{array}{cccc} & \underset{s\in S}{\text{maximize}} & & \overset{\Psi }{\overset{︷}{\theta f_{1}\left(s\right)+\left(\theta -1\right)f_{2}^{*}\left(s\right)}} \end{array}$$

The Pareto front can be obtained by solving problem Equation (5) repeatedly, assigning values to θ∈[0,1]. As mentioned, it is a set of non-dominated solutions, that is solutions where the value of $f_{1}$ cannot be improved without worsening the value of $f_{2}$, and *vice versa*. Examples in Section 5.2 show the Pareto front obtained in our lunar survey example. The next section describes our optimization model based on ${\text{ACO}}_{R}$.

## 4. Optimization Methodology

ACO is a metaheuristic for combinatorial optimization (*i.e.*, discrete domain problems). It is inspired by the foraging behavior of real ants, proposed by Dorigo *et al.* [20]. In ACO, ants build candidate solutions while exploiting search experiences and problem knowledge, represented by coefficients (pheromones and heuristic information, respectively) associated with each solution. Once an ant completes a new solution (at each iteration of the algorithm), the pheromones are updated to direct the ants towards the most promising regions of the search space. These algorithmic activities, namely (1) *construction of ant-based solutions* and (2) *pheromone updating*, are the main constructs of the ACO. ${\text{ACO}}_{R}$ [19] also maintains this structure, but adapts it to the continuous domain of the variables. Basically, the idea underlying ${\text{ACO}}_{R}$ is the shift from a discrete probability distribution to a continuous distribution to generate new solutions.

**Figure 4:**
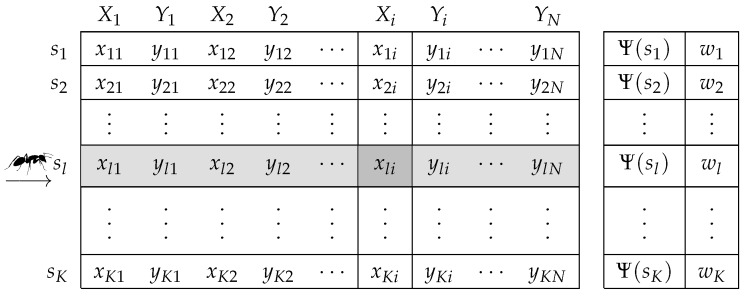
Solution archive (*T*) used by ${\text{ACO}}_{R}$ in the placement optimization problem.

${\text{ACO}}_{R}$ uses a *solution archive* (T) to store former solutions (see Figure 4) and their corresponding pheromone information. Each solution in *T* is a real-valued 2N-dimensional vector $s_{i}={\left\{x_{i},y_{i}\right\}}_{i=1\ldots N}$ corresponding to coordinates of the *N* nodes in $X\;=\;\{\left(x,y\right)\in R^{2}:x_{\text{min}}\leq x\leq x_{\text{max}},\;y_{\text{min}}\leq y\leq y_{\text{max}}\}$. A solution is an *N*-bivariate vector in $X^{N}$, which stores site positions. New solutions are obtained as samples of a continuous random vector ${\left\{X_{i},Y_{i}\right\}}_{i=1,\ldots ,N}$ whose distribution function is determined from the previous solutions and their pheromone level. This process is explained in the next section.

### 4.1. Initialization

**Figure 5:**
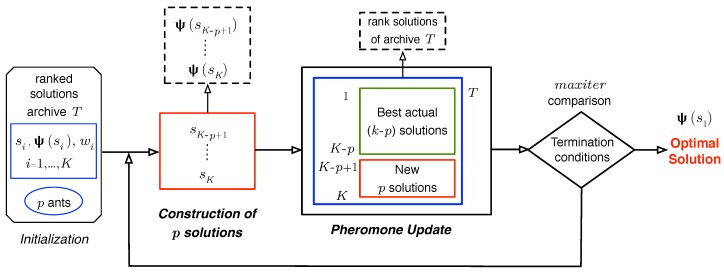
${\text{ACO}}_{R}$ main loop scheme.

Our placement optimization algorithm begins by initializing the solution archive with *K* solutions ${\left\{s_{l}\right\}}_{l=1...K}$, generated randomly. These solutions are ranked in *T* ($s_{1}$ the best and $s_{K}$ the worst) according to their objective function value, in our case expressed by Ψ in Formula (5). Besides, each solution has an associated *weight*, which is computed as:

(6) $$w_{l}=\frac{1}{qK\sqrt{2\pi }}\;e^{-\frac{{\left(l-1\right)}^{2}}{2{\left(qK\right)}^{2}}}$$

where *l* is the rank of solution $s_{l}$. The operational parameter *q* modulates the chance of selecting each row in *T*. In other words, when *q* is small, the best-ranked solutions are strongly preferred, and when it is large, the probability becomes more uniform. Since the *w*’s are used by the ants to make probabilistic decisions on how to sample the search space, they are providing the *heuristic information* of the algorithm. Initialization is the first step of the algorithm, as shown in Figure 5. At this point, we also set *p* number of ants, each of which is in charge of constructing a complete solution at each iteration.

### 4.2. Construction of Ant-Based Solutions

To compose a new solution, a sample for each *i*-th random component $X_{i}$ and $Y_{i}$ (Figure 4) is computed. The distribution function of $X_{i}$ is given by:

(7) $$F_{i}^{X}\left(x\right)=\sum_{r=1}^{K}w_{r}F_{ri}^{X}\left(x\right)$$

where $F_{ri}^{X}$ are Gaussian random variables:

(8) $$F_{ri}^{X}\left(x\right)∼N\left(\mu_{ri}^{X},\sigma_{ri}^{X}\right)$$

the means of which become $\mu_{ri}^{X}=x_{ri}$, r=1,...,K and $\sigma_{ri}^{X}$ are their standard deviations. The elements $w_{r}$ are the weights Equation (6) associated with each ranked solution in archive *T*. $Y_{i}$ components are obtained similarly.

A simple procedure to generate samples of random variables of the type in Equation (7) is the composition method [55]. First, a row *l* in *T* is selected. Each row *l* has a probability $p_{l}=w_{l}/\sum_{r=1}^{K}w_{r}$ in this selection. Then, for each i=1,...,N, sample functions $N(x_{li},\sigma_{li}^{X})$ and $N(y_{li},\sigma_{li}^{Y})$.

The standard deviations of these Gaussians functions are:

(9) $$\sigma_{li}^{X}=\xi \sum_{1\leq r\leq K}\frac{|x_{ri}-x_{li}|}{K-1}\;$$

which is the average distance between the coordinate value $x_{li}$ (*i*-th node) of solution $s_{l}$ and the values of the respective coordinate of the other solutions in *T* multiplied by *ξ*. The standard deviations $\sigma_{li}^{Y}$ are computed similarly. Parameter *ξ* regulates the tendency of the ants to explore locations that have not yet been evaluated. The process of choosing a row and building a new candidate solution is repeated *p* times (corresponding to the number of *ants*) per iteration. Before the next iteration, the algorithm updates the solution archive, as we explain in the next section.

### 4.3. Pheromone Updating

Pheromone information is used by ants to reinforce promising solutions and bias the probabilistic decisions of other ants toward these solutions. As we mentioned earlier, pheromone is stored in *T*, and its content is updated at each iteration of the algorithm. This update is accomplished by adding the *p* newly-generated solutions to the solution archive *T* and removing the same number of worst solutions (see Figure 5). Solutions in *T* are then ranked before a new iteration is started. This process ensures that only the *K* best solutions are kept in the archive, so that they effectively guide the ants in the search process.

Eventually, the algorithm stops executing solution generation cycles when no improvements for the highest-ranked solution are found after a given number of iterations (maxiter).

## 5. Placement Algorithm Evaluation

### 5.1. WSN Planning Preliminaries and Lunar Target Area Selection

**Figure 6:**
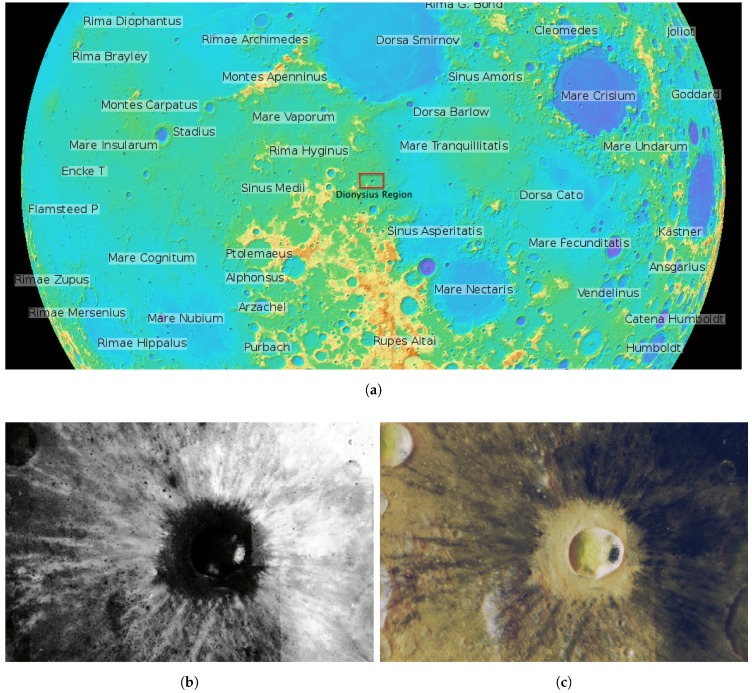
Dionysius region of interest with coordinate values S 1.6 N4.2 and W15 E 19 in degrees. (**a**) NASA’s Lunar Reconnaissance Orbiter (LRO) Wide Angle Camera (WAC) relief image in orthographic projection of the lunar near side and the Dionysius region in the center. Source: http://wms.lroc.asu.edu/lroc; (**b**) percentage of TiO_2_ weight (wt%) using the Lucey *et al.* [57] method. Source: http://www.lpi.usra.edu/lunar/tools/clementine/; (**c**) the Dionysius region of interest with an overlay of TiO_2_ percentage in black tones.

Among the different candidate lunar areas, we propose a closer study of the Dionysius region [56] by means of a WSN. The Dionysius region is located near the western edge of Mare Tranquillitatis (see Figure 6a) and is centered on the Dionysius crater ($2.8^{\circ }$N, $17.3^{\circ }$E), which has a diameter of 18 km. This region is known to have a high concentration of ilmenite material (FeOTiO${}_{3}$), which is thought to contain ${}^{3}$He (Zheng *et al.* [15]).

Our aim is to deploy the WSN at the points where models have predicted a greater abundance of ${}^{3}$He. We assume that trustworthy points correspond to zones with TiO${}_{2}$ abundance (see Section 1), a good tracer of ilmenite. Therefore, a digital map of TiO${}_{2}$ content represents an indicator function corresponding to the presence of ${}^{3}$He and can potentially be used for our proposal.

We used an image map of TiO${}_{2}$ abundance in our area of interest, delimited by latitude S$\;1.6^{\circ }$ N$\;4.2^{\circ }$ and longitude W$\;15^{\circ }$ E$\;19^{\circ }$ and generated through the Clementine Mapping Project of the Lunar and Planetary Institute (LPI) [58] web service. The image size is 1213×789 pixels, and its scale is 0.1 km/pixel in single cylindrical projection (*plate carrée*) [59] corresponding to an approximate surface of 120×78 km${}^{2}$. The weight percent (wt%) of TiO${}_{2}$ is computed based on the method described by Lucey *et al.* [57], as shown in Figure 6b, where brighter tones indicates higher Ti content (*i.e.*, higher importance or estimated ${}^{3}$He). For the convenience of this work, the original RGB Figure 6b was color inverted and indexed, such that each pixel in the image has an associated value, ranging from zero for the whitest areas (minimum TiO${}_{2}$ abundance) to 255 for the darkest areas (maximum TiO${}_{2}$ abundance). It represents our importance function, as defined in Section 3, indicating the expected ${}^{3}$He distribution at each location. Figure 6c shows the resulting indexed image overlying a relief image of the same coordinates in simple cylindrical projection obtained from the Lunar Reconnaissance Orbiter Camera [60].

Without loss of generality, we can make the following practical considerations for the deployment:

- Excluding the centered Dionysius crater, the region of deployment is smooth enough to be considered a flat surface (*i.e.*, it is not rugged). Although there may be some ${}^{3}$He inside the crater, the amounts are small and distant from other parts of the scenario and, thus, can be ignored.
- The maximum number of sensor nodes has been restricted to N=150, because spacecraft payload capacity is always limited [61]. In order to scatter these nodes in our huge target area, parameter $r_{t}$ needs to be adjusted. In our tests, we have set a long transmission range $r_{t}=6$ km.
- Antennas are assumed to be omnidirectional dipoles placed at sufficient height above the Moon surface to ensure that signal propagation (reflection, diffraction, penetration, *etc*.) is not affected by ground effects. Under these conditions, the propagation model on the lunar surface could be approximated to the *free-space* model, even for long-range distances [62,63].
- We assume that the transmission power of our nodes may be adjustable between 0dBm and 20dBm; we also assume a carrier frequency of 900MHz. This frequency allows reduced antenna dimensions of 8.32cm, which are suitable and easy to manage in space applications and also require less energy consumption than higher operation frequencies.
  An estimation of the received power at a 6-km distance can be computed using the well-known Friis equation [64].
  For instance, if we select a transmitting power of 10dBm (assuming typical dipole gains $G_{r}\;=\;G_{t}\;=\;2.15$ and a system loss factor of L=1), then we obtain a received power of −92.8dBm. Commercial transceivers of these characteristics are easily available [65].
- The sensing range is set to $r_{s}\;=\;1.5\;$ km (15 pixels in Figure 6c).
- The deployment of nodes on the lunar surface could be achieved using a rover, navigating the lunar surface. This scheme would allow controlled positioning of the nodes, although it might take a long time to put all of the nodes in place. Possible alternative methods include dropping the nodes from a spacecraft or launching them from a rover (Sanz *et al.* [66]).

### 5.2. Validation Tests

Based on adjustment tests in the deployment region, the following ${\text{ACO}}_{R}$ parameters were selected:

- Number of solutions within archive *T*: K=300.
- Number of ants: p=8.
- q=0.025 and ξ=0.65.
- maxiter=70,000.

**Figure 7:**
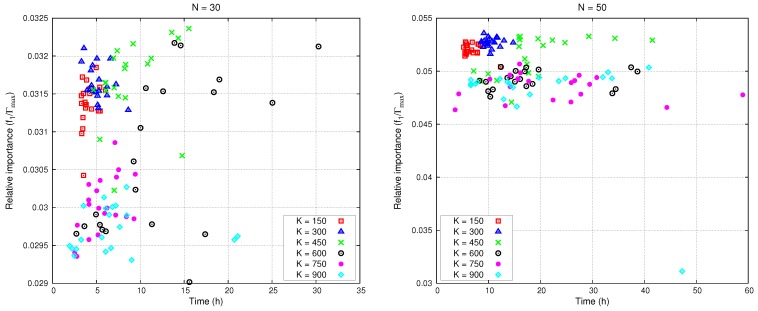
Relative importance sensed and computing time.

The better the tuning of ${\text{ACO}}_{R}$, the better the algorithm will perform (higher objective function and lower computation time). For instance, the size *K* of the solutions table is critical because it determines the complexity of the pdfs that the ants have to sample to generate new solutions. This parameter has been tested for different network sizes (N=10,30,50,70,90,110,130,150). Figure 7 shows the relative importance for N=30 and N=50. The relative importance is defined as the ratio of the importance map covered Equation (3) to the total importance ($\Gamma_{\text{max}}$) contained in the map,

(10) $$f_{1}/\Gamma_{\text{max}}=f_{1}/\int_{X}\alpha \left(x\right)dx$$

Results for K=300 have the highest relative importance and the smallest computing time of the algorithm. All computations were performed on an 8-CPU Xeon E5 computer with 128 Gb of RAM. Table 1 and Table 2 collect WSN deployment and ${\text{ACO}}_{R}$ operating parameters, respectively.

**Table 1:** Technical parameters.

number of nodes	N≤150
transmission range	$r_{t}=6$ km
sensing range	$r_{s}=1.5$ km
transmitter power	$P_{t}=10$ dBm

**Table 2:** ${\text{ACO}}_{R}$ initialization parameters.

*T* size	K=300
number of ants	p=8
heuristic parameter	q=0.025
pheromone evaporation rate	ξ=0.65
termination condition	maxiter=70000

Next, we contrast our results with those obtained using a reference heuristic used previously in Rebai *et al.* [42], which for convenience we call *four-directional placement* (FDP). For this FDP heuristic, a grid is considered over the target area, with an $r_{t}/2$ space lattice. The FDP is an iterative algorithm, which starts at a random position. At each step, it selects the adjacent, previously unselected point of the grid with the highest importance, such that the network remains connected. Following the up, down, right and left directions, the adjacent points are evaluated at $r_{t}/2$ and $r_{t}$ distances from the current position. If several points have the same value, FDP chooses one at random. During this process, the points evaluated are kept in a sorted table (observed points table) in descending order of relevance. The top one is selected as the node position, and the process continues from this point. Note that this algorithm guarantees full connection of the network.

**Figure 8:**
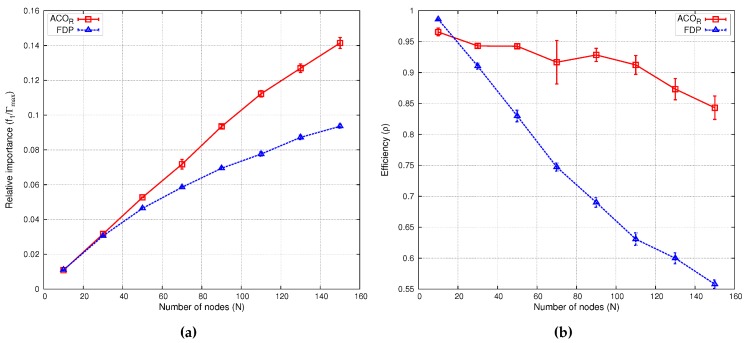
Performance comparison of ${\text{ACO}}_{R}$-based *versus* the four-directional placement (FDP) heuristic.

In our target area of Figure 6b, we considered the deployment of N=10,30,...,150 sensors and θ=1, evaluating the relative importance. These experiments were repeated 20 times (8×20 deployment simulations) for each of these algorithms, ${\text{ACO}}_{R}$ and FDP. The relative importance is displayed in Figure 8a and the efficiency in Figure 8b, with a confidence level of 95%.

**Figure 9:**
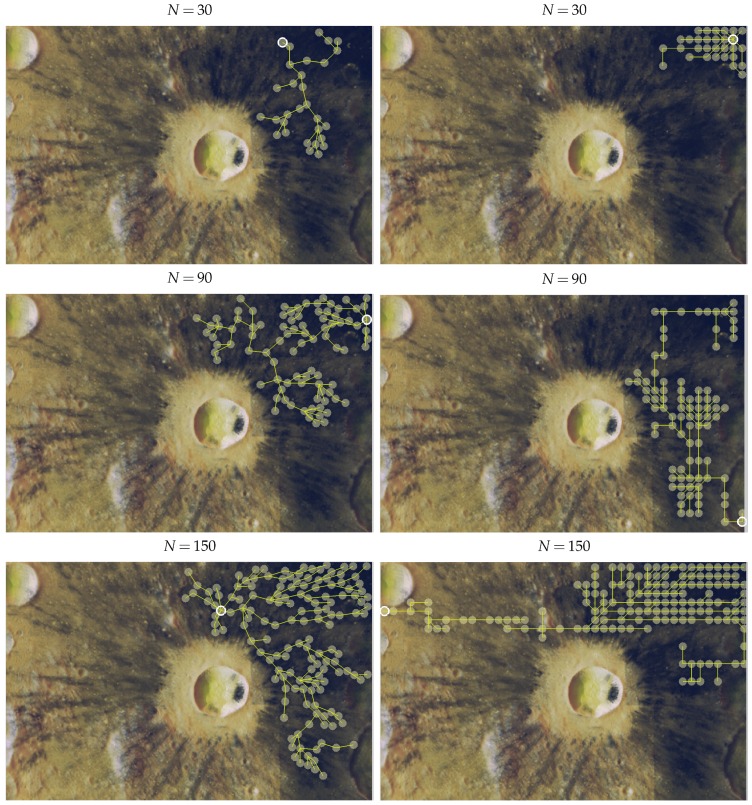
Node placement examples for different numbers of nodes in the Dionysius region. ${\text{ACO}}_{R}$ (left) and FDP (right). Numerical results in Table 3.

Efficiency *ρ* is computed as joint-coverage $\left(f_{1}\right)$ divided by the maximal information that can be sensed by *N* nodes. That is, $\rho =f_{1}/\left(N\pi r_{s}^{2}v_{max}\right)$, where $v_{max}=255$ is the maximum value of importance assigned to a point on the map. Efficiency provides insight into the quality of the deployment. Clearly, efficiency decreases with the number of nodes, since as network size increases, more nodes are used to gather less important data or simply to convey information from distant zones. Table 3 shows the maximum joint-coverage and the efficiency for different deployment instances, some of which (N=30,90,150) are depicted in Figure 9. The sensing coverage zone of each node is represented by a semi-transparent yellow circle; yellow lines are the shortest paths between the sink node (marked in white) and each node. Note that with ${\text{ACO}}_{R}$, sensing zones do not overlap in order to maximize $f_{1}$.

**Table 3:** Maximum joint-coverage in deployments.

	Relative Importance ($f_{1}/\Gamma_{\text{max}}$)		Efficiency (*ρ*)	
N nodes	${\text{ACO}}_{R}$	FDP	${\text{ACO}}_{R}$	FDP
10	0.0110	0.0110	0.9919	0.9883
30	0.0321	0.0312	0.9568	0.9328
50	0.0535	0.0489	0.9581	0.8762
70	0.0741	0.0600	0.9478	0.7669
90	0.0947	0.0742	0.9415	0.7377
110	0.1150	0.0847	0.9356	0.6892
130	0.1346	0.0919	0.9265	0.6323
150	0.1533	0.0972	0.9142	0.5800

The results of Figure 8 demonstrate that our ${\text{ACO}}_{R}$-based algorithm outperforms the FDP heuristic, even in scenarios with few nodes. These figures show how with ${\text{ACO}}_{R}$, joint-coverage $\left(f_{1}\right)$ grows steadily as the number of nodes increases. When the network is small (N≤20), the efficiency of ${\text{ACO}}_{R}$ and FDP is comparable. However, as the network size increases, ${\text{ACO}}_{R}$ maintains a high *ρ*, even for complex networks (e.g., 85% at N=150), but FDP efficiency decreases steadily.

**Figure 10:**
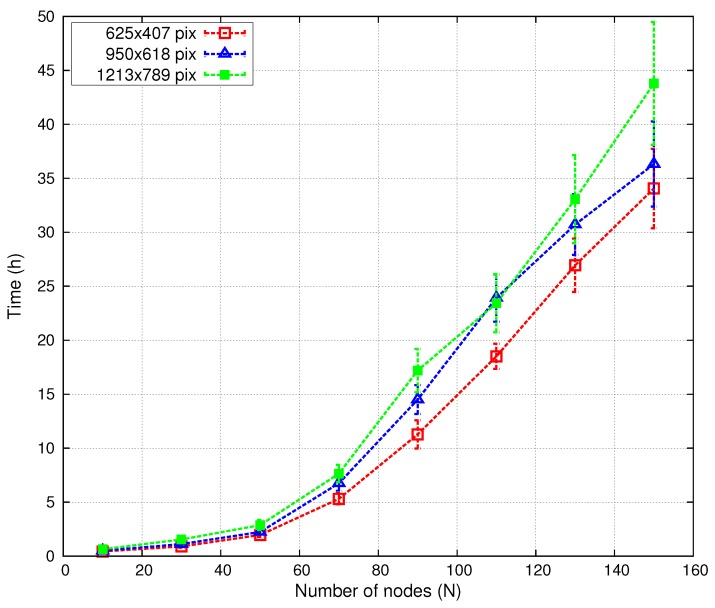
Convergence time *versus* number of nodes (*N*) and scenario size.

Figure 10 depicts the evolution of the algorithm convergence time *versus* the number of nodes and map size in both scenarios. We performed 50 tests with three different scenario sizes: 625×407, 950×618 and 1213×789 pixels (reducing the original map size). As expected, increasing the number of nodes raises the convergence time, which grows almost exponentially for N≤90, but linearly for larger network sizes. Furthermore, results for N>80 have a higher variance in both scenarios.

**Figure 11:**
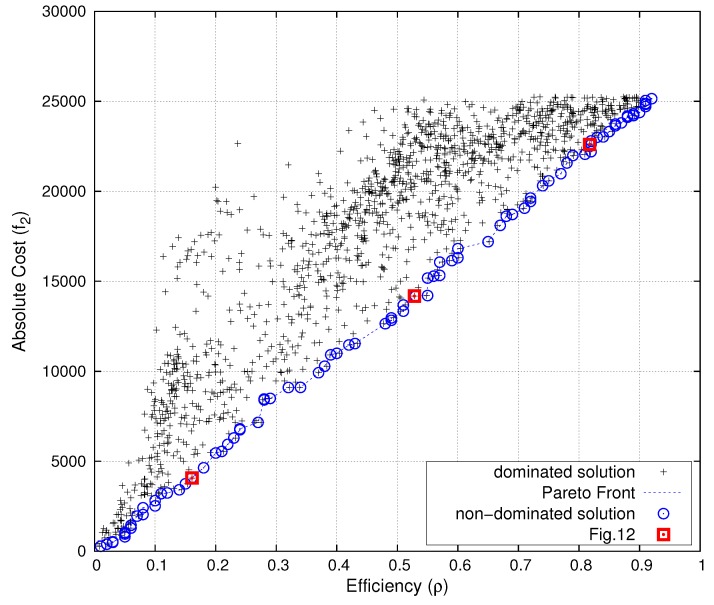
Pareto front (in blue) of $f_{1}$ (network sensing coverage) *vs*. $f_{2}$ (network cost). Deployments of Figure 12 in red.

Figure 11 shows the results of the Pareto front of functions $f_{1}$ and $f_{2}$.

The solver was executed 2040 times, from different initial positions, selected at random. Figure 11 shows a subset of representative solutions of varying parameter *θ*. The blue line shows the “best solutions”, in the sense that it is impossible to improve one of the goals without worsening the other.

**Figure 12:**
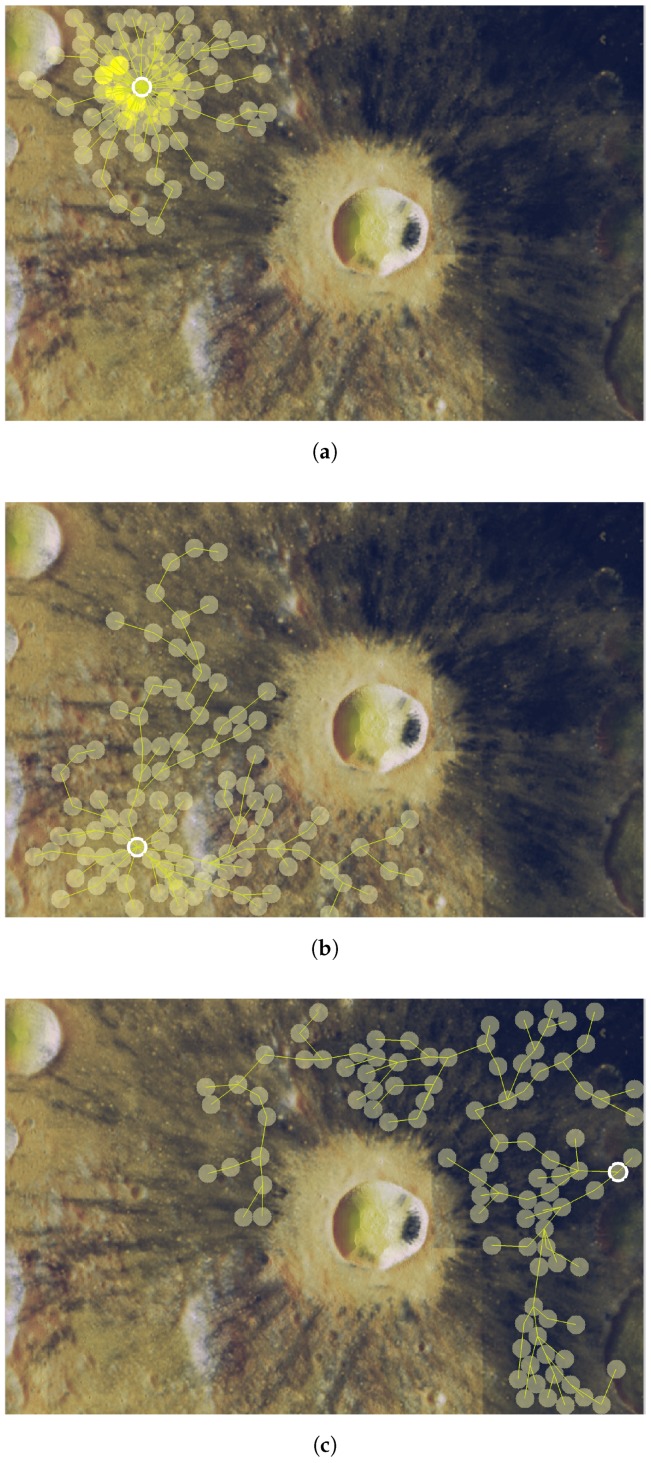
Sensing coverage of TiO_2_ content in the Dionysius region of interest. (**a**) *θ* = 0.0025, *f*_1_/Γ_max_ = 0.1608 and *f*_2_ = 4067; (**b**) *θ* = 0.05, *f*_1_/Γ_max_ = 0.5282 and *f*_2_ = 14179; (**c**) *θ* = 0.36, *f*_1_/Γ_max_ = 0.8176 and *f*_2_ = 22607.

The result reveals that the Pareto front approach is useful. The solution shows the tradeoff between cost and joint-coverage of the network. Cost is related to the energy consumed by the network during lunar nights (periods without direct sun exposure). Therefore, mission planners could compute battery consumption during lunar nights, combine this with other costs (variables of the mission), such as battery weight, performance, durability, and so on, and obtain optimal positions in terms of expected ${}^{3}$He abundance. Besides, the Pareto front results are linear, showing that there is an inverse proportional relationship between both optima magnitudes. Finally, Figure 12 also displays three sensor deployments in our region of interest for three choices of θ=(0.0025,0.05,0.36) matching the red points on the Pareto frontier represented in Figure 11. This shows how several “optimal” solutions may behave distinctly, depending on the prioritized variable in the tradeoff balance.

## 6. Conclusions

We have developed a methodology based on the ${\text{ACO}}_{R}$ metaheuristic for sensor node placement and tested this algorithm against the FDP heuristic. ${\text{ACO}}_{R}$ outperformed FDP in all of the tests. The time consumed by the algorithm scales well with the size of the search space (number of nodes and map size).

We have also evaluated a concept mission for the deployment of the proposal in a lunar scenario. In the case of relatively large network sizes (most likely in missions of this kind), efficiency *ρ* was also superior with our ${\text{ACO}}_{R}$-based algorithm.

The methodology developed in this paper can be easily extended to other complex placement problems, for example by including mass limitations or deployment time in the multi-objective function.
